# Navigating the straits: realizing the potential of proton FLASH through physics advances and further pre-clinical characterization

**DOI:** 10.3389/fonc.2024.1420337

**Published:** 2024-07-03

**Authors:** John D. Fenwick, Christopher Mayhew, Simon Jolly, Richard A. Amos, Maria A. Hawkins

**Affiliations:** ^1^ Department of Medical Physics and Biomedical Engineering, University College London, London, United Kingdom; ^2^ Department of Physics and Astronomy, University College London, London, United Kingdom; ^3^ Clinical Oncology, Radiotherapy Department, University College London NHS Foundation Trust, London, United Kingdom

**Keywords:** FLASH, proton, radiotherapy, ridge filter, adaptation, time-structure

## Abstract

Ultra-high dose-rate ‘FLASH’ radiotherapy may be a pivotal step forward for cancer treatment, widening the therapeutic window between radiation tumour killing and damage to neighbouring normal tissues. The extent of normal tissue sparing reported in pre-clinical FLASH studies typically corresponds to an increase in isotoxic dose-levels of 5–20%, though gains are larger at higher doses. Conditions currently thought necessary for FLASH normal tissue sparing are a dose-rate ≥40 Gy s^-1^, dose-per-fraction ≥5–10 Gy and irradiation duration ≤0.2–0.5 s. Cyclotron proton accelerators are the first clinical systems to be adapted to irradiate deep-seated tumours at FLASH dose-rates, but even using these machines it is challenging to meet the FLASH conditions. In this review we describe the challenges for delivering FLASH proton beam therapy, the compromises that ensue if these challenges are not addressed, and resulting dosimetric losses. Some of these losses are on the same scale as the gains from FLASH found pre-clinically. We therefore conclude that for FLASH to succeed clinically the challenges must be systematically overcome rather than accommodated, and we survey physical and pre-clinical routes for achieving this.

## 1 Introduction

Normal tissue volumes irradiated to high and intermediate dose-levels during radiotherapy (RT) have been substantially reduced using intensity-modulated, image-guided and proton beam technologies (1–3). Despite this, organs adjacent to tumours continue to receive high dose-levels, leading to serious toxicities and limiting prescribed doses (4, 5). A fundamental advance is therefore needed to lessen normal tissue damage.

FLASH RT delivered at ultra-high dose-rates (UHDR) has been hailed as a potential paradigm shift in cancer treatment (6). In pre-clinical experiments in the 1960s, substantially less normal tissue damage was observed when radiation was delivered at ultra-high rather than standard dose-rates (7). A further pre-clinical study in 2014 found that tumour growth was suppressed similarly by irradiation at both dose-rates, and thus the therapeutic window was wider at UHDR (8). Over the last decade these findings have been confirmed in many pre-clinical experiments carried out for multiple tumour types and normal tissues.

Broadly, the FLASH effect is seen at radiation dose-rates ≥40 Gy s^-1^ when doses of 5–10 Gy or higher are delivered in durations ≤0.2–0.5 s (7, 9, 10). In these circumstances doses can be raised by 0–60% above levels delivered at standard dose-rates of 1–2 Gy per minute, without increasing normal tissue damage. These increases in isotoxic dose-levels result from FLASH sparing and are denoted here succinctly as ‘FLASH gains’. Gains are typically 5–20% but larger for doses ≥20 Gy (11).

To date, isochronous and synchro cyclotron proton accelerators are the only clinical RT systems that have been adapted to irradiate deep-seated tumours at ≥40 Gy s^-1^. Synchrotron systems are presently being adapted but require more modification since they standardly generate maximum average beam currents of 5–32 nA, whereas isochronous cyclotrons generate 300–800 nA (12, 13). These currents compare to ~80–600 nA needed to deliver 40 Gy s^-1^ to volumes of 125–1000 ml, ignoring beam losses in scattering systems and dead-times between spot deliveries in scanned fields (13). FLASH dose-rates have also been achieved using a 7 MV X-ray beam generated by an experimental super-cooled linear accelerator (linac) (14). And a patient’s skin lesion has been treated at 167 Gy s^-1^ using a 5.4 MeV electron beam, but electrons of this energy are only suitable for treating superficial tumours (7).

**Figure 1:**
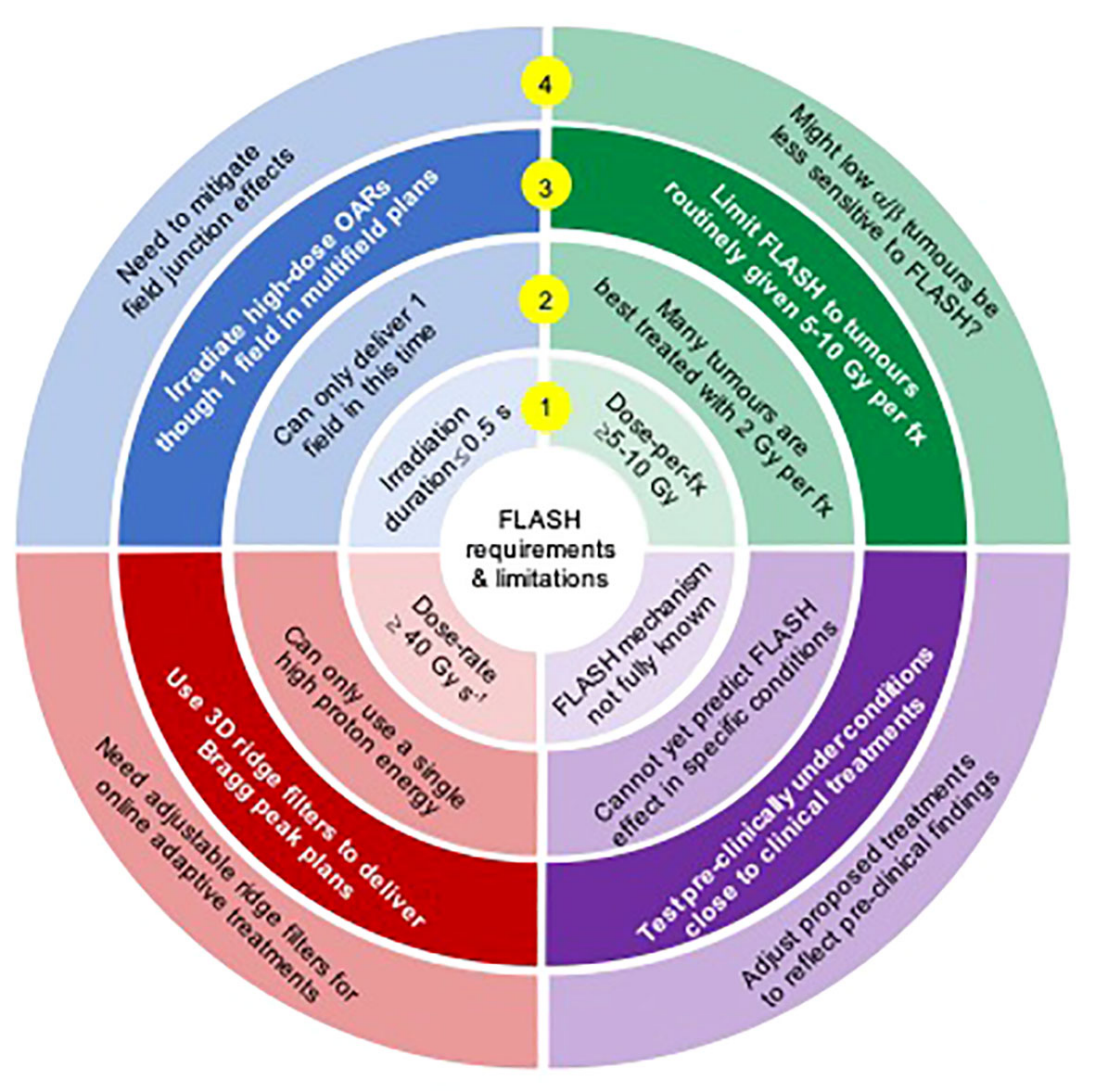
A summary of FLASH requirements (inner ring 1), resulting challenges for proton FLASH (ring 2), potential solutions (ring 3) and issues arising from the solutions (ring 4).

Even for cyclotron systems, the requirements for UHDR, short delivery times and moderately high doses-per-fraction present tough challenges for delivery of FLASH proton beam therapy (PBT), as outlined in **Figure 1**. Left unaddressed, these will lead to several treatment delivery compromises. In the *Challenges* section of this review we describe these compromises together with associated dosimetric losses, which have the potential to dissipate or overwhelm the FLASH gains. Indeed, no clear advantage was seen for FLASH-RT in the first patient treated, who had two lymphoma skin lesions, one given regular dose-rate RT and the other FLASH electrons (7). We therefore propose that the challenges must be systematically met for the full potential of FLASH to be clinically realized, and in the *Solution*s section of the review we describe how this might be accomplished.

## 2 Challenges for proton FLASH

### 2.1 Physics challenges

#### 2.1.1 Bragg peak delivery

Standard energy-switching cannot be used to deliver proton Bragg peaks to multiple tissue layers in FLASH treatments, because of beam losses (13) and the time overhead in changing the beam energy extracted from the cyclotron, which can be between 80 ms and several seconds per change (15). The first clinical implementation of FLASH-PBT has therefore used a transmission beam of high-energy protons which passes right through patients (16). But this loses a key advantage of PBT, that protons with well-chosen energies stop just after the tumour.

Using 3D ridge filters, proton ranges can be varied to match Bragg peak locations to target volumes without changing the beam energy extracted from the cyclotron. The filters provide levels of proton range compensation and modulation that vary spatially across fields. Design principles for these devices have been described, along with 3D printing methods for their construction and software to compute the dose-distributions they generate (17–19).

Transmission and Bragg peak FLASH plans have been compared for eight patients with recurrent head-and-neck cancer: three oropharynx, two oral cavity, one nasopharynx, one sinonasal, one salivary gland (20). Differences in calculated organ-at-risk (OAR) doses are summarized in **Table 1**. Mean doses to the oral cavity, left/right cochlea and left/right parotid glands were on average 38.0% higher in the transmission plans. Maximum doses to the oral cavity, mandible, spinal cord, brainstem, chiasm and left/right optic nerves, cochlea and parotids were on average 26.5% higher in transmission plans, although maximum doses to the left/right lens were on average 26.9% lower.

**Table 1:** Increases in OAR dose-metrics in transmission versus Bragg peak proton plans, and in unadapted versus adaptive treatments.

Study	Metric	OAR	Increase (%)
Pennock et al., 2023 (20)	Maximum dose	Oral cavity	+5.7
Head-&-neck, 6 Gy/fx,		Mandible	+23.6
Transmission vs Bragg peak		Spinal cord	+75.3
		Brainstem	+19.2
		Chiasm	+9.6
		Left optic nerve	+23.3
		Right optic nerve	+15.1
		Left cochlea	+23.5
		Right cochlea	+31.2
		Left parotid	+44.1
		Right parotid	+20.5
		Left lens	-0.5
		Right lens	-55.3
	Mean dose	Oral cavity	+6.6
		Left cochlea	+32.6
		Right cochlea	+64.9
		Left parotid	+84.6
		Right parotid	+1.3
Wei et al., 2021 (21)	Maximum dose	Oesophagus	+22.9
Lung, 18 Gy/fx,		Spinal cord	+55.0
Transmission vs Bragg peak		Heart	-5.4
	V_7 Gy_*	Lung - GTV	+68.9
Bobic et al., 2021 (22)	D_1cc_**	Spinal cord	+38.4
Head-&-neck, 70 Gy/31–35 fx,		Brainstem	+20.2
Unadapted vs adaptive	Mean dose	Oral cavity	+0.8
		Contralateral parotid	+16.3
		Ipsilateral parotid	+24.4
		Larynx	+16.7

* Fractional OAR volume receiving ≥7 Gy.

** Minimum dose to most highly irradiated 1 cc of OAR

The shading breaks up the results by study and metric.

**Table 1** also includes results from a similar study for ten patients with lung cancer who received proton stereotactic ablative body RT (SABR) (21). Volumes of lung minus gross tumour volume (GTV) receiving ≥7 Gy were 68.9% higher in transmission than Bragg peak plans. Maximum doses to the oesophagus and spinal cord were on average 39.0% higher in the transmission plans, though maximum heart doses were 5.4% lower. Overall, then, OAR irradiation was considerably reduced in Bragg peak compared to transmission plans.

#### 2.1.2 Achieving FLASH dose-rates

In the first trial of proton FLASH, *FAST-01*, bone metastases were treated using single transmission fields (16). The fields were composed of uniformly weighted 250 MeV protons pencil beams generated by a FLASH-enabled *ProBeam* system based on an isochronous cyclotron (Varian Medical Systems, Palo Alta, CA). In the trial’s dosimetry workup, pencil beam scanning (PBS) dose-rates *R_PBS_* up to 61.7 Gy s^-1^ were achieved at 5 cm depth in a 5×12 cm^2^ field using nozzle currents up to 160 nA (23, 24). Thus, the system surpassed the FLASH dose-rate threshold of 40 Gy s^-1^.

Treatments built from multiple intensity-modulated transmission proton fields were planned for seven patients with hepatocellular carcinoma (median internal CTV, 78.2 cc; range, 9.2–169.2 cc) (25). The prescribed dose was 67.5 Gy in 4.5 Gy fractions. Plans were created for a *ProBeam* system delivering 245 MeV protons with 165 nA beam current, setting a minimum spot time of 0.5 ms. Average dose-rates *R_ave_* in 5-field plans exceeded 40 Gy s^-1^ in ~80–95% of the volumes of oesophagus, stomach, kidneys, chest, heart wall and liver excluding GTV that received the prescribed dose or greater (25, 26).

Dose-rates have also been calculated for FLASH plans comprising multiple intensity-modulated Bragg peak fields generated using ridge filters. In one study, FLASH treatments giving 5 Gy per fraction were optimized for three lung cancer patients, two with tumours of diameter 4.5 and 5.2 cm and one with a recurrence in a lymph node. For 300 nA beam current and 1 ms minimum spot duration, the dose-per-fraction and dose-averaged dose-rate, *R_DADR_*, simultaneously exceeded 4 Gy and 40 Gy s^-1^ in ~40–70%, 70–100% and 100% of lung, heart and oesophagus regions lying within 5 mm of the planning target volume (PTV) (27, 28). In another study, single fraction FLASH treatments of 6 and 10 Gy were planned for eight patients with recurrent head-and-neck cancer (20). The plans used a *ProBeam* model, specifying 215 nA beam current and 2 ms minimum spot duration. Average fractional volume coverage with *R_DADR_* ≥40 Gy s^-1^ was >90% for all normal structures analysed, with ~5% receiving 20 Gy s^-1^ or less.

These planning studies show that cyclotron proton accelerators can deliver FLASH dose-rates to substantial percentages of highly-irradiated normal tissues. However, dose-rates fell below the FLASH threshold in small sub-volumes. Furthermore, several measures of dose-rate were used, and it is not yet known how they correspond to measures used in pre-clinical experiments (see Section 2.2.2.). Notably, the *R_ave_* calculations did not include time intervals between deliveries of individual fields in multi-field plans, and *R_DADR_* does not account for these intervals or dead-times between spot deliveries. Inclusion of these intervals would reduce the calculated dose-rates.

#### 2.1.3 Delivering several fields per fraction

Routine treatments deliver fields from multiple angles, to spread out radiation doses amongst different normal tissues. Usually all or several fields are delivered at each treatment fraction, as this is advantageous according to linear-quadratic (LQ) modelling (29). However, the slow gantry rotation speed and need to change ridge filters make it difficult to deliver more than one field without exceeding the irradiation duration limit for FLASH sparing, thought to be 0.2–0.5 s.

The effect of delivering one rather than all fields at each fraction has been analysed in a planning study of SABR treatments of lung cancer (30). Plans were built from 3–9 proton transmission fields and equivalent doses in 2 Gy fractions (EQD2s) were calculated from physical dose-distributions according to the LQ model. Mean EQD2s in the ipsilateral lung minus PTV increased when only one field-per-fraction was delivered. Increases were independent of the number of fields in a plan and required FLASH gains of ~30% to offset them, around the scale of FLASH gains found in pre-clinical studies. Thus rates of pneumonitis and lung fibrosis, which depend on lung mean EQD2, are unlikely to be much reduced by FLASH treatments that deliver only one field-per-fraction.

Normal tissue volumes receiving EQD2s greater than the prescribed dose-level were also increased by delivering a single field-per-fraction. FLASH gains required to offset these rises were 33% for 3 field plans, falling to 15% for 9 fields.

#### 2.1.4 Adaptation of Bragg peak treatments

Treatment plans are often adapted to account for dosimetrically-consequential anatomical changes seen in cone-beam CT (CBCT) images collected ahead of individual fractions. Adaptation is potentially more critical for PBT than for X-ray treatment, since anatomical variations lead to changes in proton ranges and thus targeting inaccuracies (31, 32).

In a head-and-neck proton planning study, cumulative dose-distributions that would be achieved using daily online plan adaptation were compared to those resulting from no adaptation (22). The study was carried out for ten patients with tumours located in the oral cavity, oropharynx and larynx, treated in 31–35 fractions. The CTV-PTV margin was set to zero, and consequently CTV coverage was poorer without adaptation. Differences in OAR doses are listed in **Table 1**. Doses to the most highly irradiated 1 cc of cord and brainstem were on average 29.3% higher in unadapted plans, while mean doses to the contralateral and ipsilateral parotids and larynx, constrictor muscles and oral cavity were on average 15.0% higher.

For Bragg peak FLASH-PBT it is planned to use ridge filters to achieve proton pull-back and range modulation. However, these filters presently take 1.5–12 hours to 3D print (17–19), precluding online adaptation immediately ahead of treatment fractions. FLASH adaptation could be carried out offline, for example by adjusting treatments once per week using images collected the previous day. In the head-and-neck proton planning study, much of the gain found for daily online adaptation was maintained by weekly online adaptation (22). Similar results might be expected for offline weekly adaptation, since in the online weekly approach adaptations of most treatment fractions reflect images collected on earlier days. Alternatively, a plan-of-the-day approach could be used, creating a library of plans up-front and using the one that best matches the patient geometry at each fraction (33). But this would require manufacturing and storing many ridge filters for each patient.

#### 2.1.5 FLASH beam metrology

Accurate radiation dose measurement is fundamental to the safety and efficacy of RT. Machine calibration should be traceable to a National Measurement Institute (NMI) and doses should be precisely controlled by real-time beam monitoring systems during treatment. In current dosimetry protocols, treatment beams are calibrated using ionization chambers which in turn have calibration factors traceable to an NMI (34). However, the chamber readings require correction for ion recombination and the scale of correction rises with dose-rate, adding to measurement uncertainty.

Recombination levels can be large at UHDR, substantially reducing chamber collection efficiencies if doses-per-pulse are high. For an idealized parallel plate chamber with 2 mm electrode separation and 200 V applied, collection efficiencies of 0.9994, 0.9938, 0.9412 and 0.6237 have been calculated at doses-per-pulse of 0.1, 1, 10 and 100 mGy (35). The calculations assume pulse durations much shorter than the typical 0.1 ms ion transit time across ionization chambers. Pulses generated by synchro cyclotrons meet this condition, but beams generated by isochronous cyclotrons are continuous on this timescale, improving collection efficiencies. Experimental methods for determining large recombination correction factors are not well established. Therefore it is essential to establish an accurate and traceable dosimetry chain for FLASH-PBT, both for patient safety and to allow meaningful interpretation of outcomes from clinical trials.

### 2.2 Clinical and pre-clinical challenges

#### 2.2.1 Tumour selection

For many tumours the α/β ratio, which defines fractionation sensitivity in the LQ model, is substantially larger than the 3 Gy value typical of late toxicities (36). At standard dose-rates such tumours are generally treated more effectively using 2 Gy dose-per-fraction rather than the ≥5–10 Gy thought necessary to achieve normal tissue sparing at UHDR. For some tumours, though, it is advantageous to use doses-per-fraction ≥5 Gy routinely, either because α/β is lower (37) or because the tumour is best treated using SABR or stereotactic radiosurgery (SRS), which give high EQD2s in short treatments delivered in a few precisely delivered fractions (38, 39).

Böhlen et al. used the LQ model with typical tumour and normal tissue α/β ratios of 10 Gy and 3 Gy to determine how much greater normal tissue EQD2s were for hypofractionated schedules rather than standard 2 Gy schedules giving the same tumour EQD2 (9). In normal tissues receiving the full prescribed dose-level, FLASH gains needed to offset the EQD2 increases were ~15% when 5 Gy per fraction was prescribed, rising to ~30% at 10 Gy per fraction. For normal tissues receiving 60% of the target dose, the FLASH gains required were half these levels.

These results indicate that much of the normal tissue FLASH sparing seen pre-clinically at ≥5–10 Gy per fraction is undone if these doses-per-fraction are prescribed to high α/β tumours that are best treated in 2 Gy fractions. To achieve an overall benefit from FLASH schedules giving ≥5 Gy per fraction, tumours should be chosen from amongst those for which benefit from such doses-per-fraction at standard dose-rates: tumours treated using SABR/SRS or with low α/β.

#### 2.2.2 Dose-rate definition

Proton beams generated by isochronous cyclotrons are essentially continuous. However, pencil beam scanning lends a pulsatile aspect to irradiation since each tissue element receives dose contributions from several pencil beams delivered at different times, with individual contributions reflecting pencil beam weights and spot locations relative to the tissue element. Pulse durations are on the order of milliseconds, determined by delivery times of individual pencil beams.

Several measures have been proposed to describe effective FLASH dose-rates for these irregular pulsatile deliveries. The simplest is the average dose-rate *R_ave_* (26), defined as the total dose-per-fraction *D_tot_* delivered to a tissue element, divided by the interval *T* between the first and last times at which the element is irradiated during the fraction. Alternatively, the pencil beam scanning dose-rate *R_PBS_* (24) excludes dose contributions below a small threshold $D_{th}$ at the beginning and end of irradiation of the tissue element, thus


(1) $$R_{PBS}=\left(D_{tot}-2D_{th}\right)/\left(T_{1}-T_{0}\right)$$

In **Equation (1)** *T_0_* and *T_1_* are the times at which the tissue element has received doses of *D_th_* and *D_tot_* – *D_th_* respectively and $D_{th}$ is often defined as a fraction of the prescription dose.

The percentile dose-rate *R_Perc_* is a variant of *R_PBS_* that defines *D_th_* as a fraction of *D_tot_* (26). Another variant is the maximum percentile dose-rate *R_MP_*, which represents the fastest rate at which a dose $D_{tot}-2D_{th}$ is delivered to the tissue element. This allows for the possibility, for example, that the interval between delivery of cumulative doses of $0.5\;D_{th}$ and $D_{tot}-1.5\;D_{th}$ might be shorter than the interval between delivery of $D_{th}$ and $D_{tot}-D_{th}$ (26).

The dose-averaged dose-rate *R_DADR_* is an earlier proposed measure. It is the mean of the individual dose-rates generated in the tissue element when the various spots are delivered, weighted by the doses the element receives from each spot delivery (28). A model-based approach has also been proposed which seeks to quantify the fraction of dose delivered to the voxel while FLASH sparing is taking place. Sparing is considered active at any time-point lying within a time-window across which the dose and average dose-rate delivered to the tissue element exceed thresholds defined in the model (40).

It has yet to be established how dose-rates calculated according to these definitions correspond to the 40 Gy s^-1^ FLASH threshold identified in pre-clinical FLASH experiments. Pre-clinical researchers have usually delivered radiation at uniform rates or in sequences of uniform pulses, and calculated dose-rates as the total dose divided by the total duration of irradiation interval. Valuable indications about the likely performance of clinical scanned beam treatments will be provided by pre-clinical experiments which deliver irregular pulsed radiation sequences, similar to those that will be used clinically (41).

#### 2.2.3 Pre-clinical FLASH testing ahead of clinical treatments

The normal tissue FLASH sparing achievable using specific doses-per-fraction, dose-rates and irradiation durations cannot yet be reliably predicted, in part because the mechanistic basis of FLASH remains unclear (42). So, when designing patient treatments it will be informative to pre-clinically test FLASH schedules with dose and time-structures close to those intended for clinical use.

**Table 2** summarizes normal tissue FLASH experiments carried out in 29 pre-clinical studies (43–71) previously tabulated (11, 72) or recently published (54, 62, 67, 69–71). The experiments are grouped by the normal tissue endpoints analysed, which range from survival through to microscopic changes at tissue and cell levels. Molecular endpoints such as DNA damage are not listed. Specific normal tissues studied were skin, intestine, pericardium, lung and brain. Clinically relevant endpoints were skin reactions, intestinal fibrosis, pericardial oedema, pneumonitis, lung fibrosis, and memory and neurocognitive changes.

**Table 2:** Summary of pre-clinical studies of normal tissue damage following FLASH irradiation. Studies are listed by endpoint, with shading used to further visualize groups of connected endpoints.

Endpoint	Species & tissue	Irradiated region*	FLASH beam-type^†^ (energy, PRF^$^)	Short reference
Survival (4 d^‡^)	Zebrafish	Whole body	p*-ic-sT* (224 MeV, NA)	Beyreuther 2019 (43)
Survival (5 d)	Mouse gut	Whole body	e (7 MeV, 400 s^-1^)	Hornsey 1971 (44)
Survival (20 d)	Mouse gut	Abdomen	e (20 MeV, NA)	Loo 2017 (45)
Survival (9, 12 d)	Mouse gut	Abdomen	p*-ic-sT* (229 MeV, NA)	Zhang 2020 (46)
Survival (23 d)	Mouse gut	Abdomen	p*-sc-sT* (230 MeV, 756 s^-1^)	Evans 2021 (47)
Survival (50, 100, 150 d)	Mouse skin	Hemithorax	e (16 MeV, 90 s^-1^)	Soto 2020 (48)
Survival (60 d)	Mouse skin	Leg	p*-ic-sT* (230 MeV, NA)	Velalopoulou 2021 (49)
Acute radiation syndrome (within 60–68 d)	Mouse	Whole body	γ*-syn* (93/124 keV, NA)	Smyth 2018 (50)
Fish length (5 d)	Zebrafish	Whole body	e (6 MeV, single pulse)	Ollivier 2021 (51)
Spinal curvature (3, 4 d)	Zebrafish	Whole body	p*-ic-sT* (224 MeV, NA)	Beyreuther 2019 (43)
Foot deformation (6 month)	Rat foot	Feet	e (7 MeV, NA)	Field 1974 (52)
Skin reactions (11–25 d)	Mouse skin	Feet	p*-ic-sN* (244/250 MeV, 52 s^-1^ **)	Sørensen 2022 (53)
Skin reactions (8 wk)	Mouse skin	Hemithorax	e (16 MeV, 90 s^-1^)	Soto 2020 (48)
Skin reactions (8 wk)	Mouse skin	Leg	γ*-Xrt* (150 kVp^††^, continuous)	Miles 2023 (54)
Skin reactions (within 8 months)	Mouse skin	Leg	p*-ic-sT* (230 MeV, NA)	Velalopoulou 2021 (49)
Skin reactions (7–35 d)	Rat skin	Feet	e (7 MeV, NA)	Field 1974 (52)
Skin reactions (7–48 wk)	Mini pig skin	Back	e (6 MeV, 100 s^-1^)	Vozenin 2019 (55)
Necrosis (7 wk)	Mouse tail	Tail	e (10 MeV, 50 s^-1^)	Hendry 1982 (56)
Intestinal fibrosis (8 wk)	Mouse gut	Upper gut	p*-ic-sT* (230 MeV, NA)	Diffenderfer 2020 (57)
Gastrointestinal syndrome (within 60–68 d)	Mouse gut	Abdomen	γ*-syn* (93/124 keV, NA)	Smyth 2018 (50)
Crypt survival (3.75 d)	Mouse gut	Abdomen	e (6 MeV, single pulse)	Ruan 2021 (58)
Crypt survival (4 d)	Mouse gut	Abdomen	e (16 MeV, 108 s^-1^)	Levy 2020 (59)
Crypt cell proliferation (3.5 d)	Mouse gut	Abdomen	p*-ic-sT* (230 MeV, NA)	Diffenderfer 2020 (57)
Pericardial edema (3, 4 d)	Zebrafish heart	Whole body	p*-ic-sT* (224 MeV, NA)	Beyreuther 2019 (43)
Lung fibrosis (8, 16, 24, 36 wk)	Mouse lung	Thorax	e (4.5 MeV, 100–150 s^-1^)	Favaudon 2014 (60)
Lung fibrosis (16–20 wk)	Mouse lung	Thorax	e (4.5 MeV, NA)	Fouillade 2020 (61)
CT-assessed pneumonitis (2, 4, 6, 9, 12 wk)	Mouse lung	Thorax	γ*-linac* (10 MV^$$^, NA)	Ren 2023 (62)
Lung cell proliferation (1 wk)	Mouse lung	Thorax	e (4.5 MeV, NA)	Fouillade 2020 (61)
Memory tests, neurogenesis (2 months)	Mouse brain	Whole body	e (6 MeV, 100 s^-1^)	Montay-Gruel 2017 (63)
Neurocognitive tests (2–6 months)	Mouse brain	Whole body	γ*-syn* (102 keV, NA)	Montay-Gruel 2018 (64)
Neurocognitive tests (10 wk)	Mouse brain	Whole body	e (16/20 MeV, 180/108 s^-1^)	Simmons 2019 (65)
Neurocognitive tests (2, 4 months)	Mouse brain	Whole body	e (6 MeV, single pulse)	Alaghband 2020 (66)
Memory, novel object recognition (4 months)	Mouse brain	Brain (2#)	e (6 MeV, single pulse)	Allen 2022 (67)
Memory, novel object recognition (2 months)	Mouse brain	Brain	e (6 MeV, single pulse)	Montay-Gruel 2019 (68)
Memory, novel object recognition (1 month)	Mouse brain	Brain (1–4#)	e (6 MeV, single pulse)	Montay-Gruel 2021 (69)
Memory, novel object recognition (1 month)	Mouse brain	Brain	e (6 MeV, 2 pulses 0.01 s apart)	Montay-Gruel 2021 (69)
Neurologic symptoms (within 38 d)	Mouse brain	Brain	γ*-syn* (93/124 keV, NA)	Smyth 2018 (50)
Social interactions, light-dark box (4 months)	Mouse brain	Brain (2#)	e (6 MeV, single pulse)	Allen 2022 (67)
Dendritic spine density (10 wk)	Mouse brain	Whole body	e (16/20 MeV, 180/108 s^-1^)	Simmons 2019 (65)
Mature/immature neurons (1 month)	Mouse brain	Whole body	e (6 MeV, single pulse)	Alaghband 2020 (66)
Synaptic structure and density (6 months)	Mouse brain	Brain (2#)	e (6 MeV, rwo pulses 2 d apart)	Allen 2022 (67)
Hippocampal cell division (2 months)	Mouse brain	Whole body	γ*-syn* (102 keV, NA)	Montay-Gruel 2018 (64)
Astrogliosis (2 months)	Mouse brain	Whole body	γ*-syn* (102 keV, NA)	Montay-Gruel 2018 (64)
Astrogliosis (1 month)	Mouse brain	Whole body	γ*-syn* (102 keV, single pulse)	Montay-Gruel 2020 (70)
Hippocampal synaptic LTP (6 months)	Mouse brain	Brain (2#)	e (6 MeV, single pulse)	Allen 2022 (67)
Hippocampal synaptic LTP (4 months)	Mouse brain	Brain (10#)	e (6 MeV, single pulse)	Limoli 2023 (71)

* Numbers of FLASH fractions tested, #, are also shown if >1.

^†^ Electron (e), proton (p) and photon (γ) beams were used. The electron beams were all generated by linacs. Proton beams were generated by isochronous cyclotrons (ic) and synchro cyclotrons (sc), and were either scattered (sT) or scanned (sN). Photons were generated using a synchrotron (syn), a kilovotage X-ray tube (Xrt) and a super-conducting linac (linac).

^$^ PRF denotes pulse repetition frequency. NA indicates PRF values not available in the literature.

^‡^ Endpoint evaluation times following irradiation are listed in days (d), weeks (wk) and months.

** The tabulated PRF was estimated from **Figure 2** of Sørensen et al., 2022 and describes the frequency of delivery of individual pencil beams.

^††^ The mean X-ray energy in the 150 kVp beam was 52.5 keV.

^$$^ X-rays were generated by colliding 10 MeV electrons into a bonded tantalum-aluminium target. The resulting photon spectrum had mean energy ~1.8 MeV.

The table lists 46 experiments in total, considering each study endpoint separately. Of these 27 used electron beams, 9 X-ray photons and 10 protons. The electron beams were generated by linacs and ranged in energy from 4.5–20 MeV. Seven photon experiments used synchrotron-generated X-ray beams of energy 93–124 keV. One used X-rays generated by an experimental high-intensity 10 MeV linac, creating a spectrum of photons with 1.8 MeV mean energy (62, 73). The remaining photon experiment used an X-ray tube to generate a 150 kVp beam with 52.5 keV mean photon energy (54).

Of the ten proton experiments, nine investigated scattered proton fields and one a scanned field (53). The latter experiment measured the dose response of skin reactions on mice legs irradiated in water at UHDR and standard dose-rates. A 2×3 cm^2^ transmission field was used, composed of pencil beams on a 5.0×5.1 mm^2^ grid. UHDR irradiations had 0.35–0.73 s durations and 65–92 Gy s^-1^ field dose-rates, defined as planned field doses divided by field irradiation times. Substantial normal tissue sparing was found at UHDR, with FLASH gains up to 58%.

Most studies listed in **Table 2** tested single fractions. However, some recent pre-clinical studies have tested SABR/SRS-like schedules giving 2, 3, 4 and 10 fractions with gaps of 1–2 days between them (67, 69, 71). As described in the *Solutions*, further pre-clinical normal tissue data are needed to more fully characterize FLASH sparing in conditions similar to treatments, including for UHDR irradiations split by time intervals, reflecting delivery of multiple fields per fraction (7, 74).

Pre-clinical studies of UHDR tumour irradiation have been reviewed (75). Experiments carried out for syngeneic models of lung carcinoma, glioblastoma, pancreas cancer, ovarian cancer, oral squamous cell carcinoma, sarcomas and breast cancer in mice were included in the review, along with studies using human xenografts of glioblastoma, breast cancer and T-cell acute lymphoblastic leukaemia. The experiments tested schedules comprising 1–5 treatment fractions, the median number being one.

Systematic analysis of results from these studies supported the isoffectiveness of UHDR and standard dose-rate tumour irradiation (75). But it was noted that the endpoint most commonly reported was change in tumour size following irradiation, which is only weakly correlated with long-term tumour control. Furthermore, numbers of tumours in each treatment group ranged from 3 to 15, limiting studies’ power to detect differential effects of FLASH irradiation.

Clinical trials that compare outcomes for the same doses given at ultra-high and standard dose-rates are likely to be influential in the adoption of FLASH RT (7). Such trials may focus on tumours routinely treated using the ≥5–10 Gy per fraction currently considered necessary for FLASH sparing, including some low α/β tumours. However, α/β is thought to be linked to proliferation, with more slowly proliferating tumours having lower α/β ratios, similar to those of slowly turning-over normal tissues (76, 77). This raises the possibility that the FLASH response of low α/β tumours might reflect that of normal tissues, and thus these tumours might be controlled less effectively at UHDR, a conjecture it would be prudent to check pre-clinically ahead of trialling.

## 3 Potential solutions for proton FLASH

### 3.1 Scale of the problem

**Table 3** summarizes the dosimetric consequences of compromises in the delivery of proton FLASH. These are contrasted with the dose increases achieved at UHDR in pre-clinical studies without exceeding toxicity levels observed at standard doses and dose-rates. The tabulated OAR dose increases that result from delivery compromises can be compared directly with the isotoxic dose-level increases achieved via FLASH sparing. Tabulated EQD2 increases have been further processed to aid comparison, as detailed in the table footnotes.

**Table 3:** Isotoxic dose increases for ultra-high vs standard dose-rates inpre-clinical studies (blue), compared to estimated dosimetric losses from potential proton FLASH delivery compromises (green).

Factor	Metric	Effect
Gain from FLASH delivery	Rise in dose deliverable without increasing toxicity	
UHDR vs standard dose-rate	Uniform OAR dose	0–60% range, 5–20% typical
Losses from delivery compromises	Rise in dose due to compromise	
Transmission vs Bragg peak	D_max_ in H&N OARs^a^,	38%
	D_max_ in lung OARs^b^	24%
	D_mean_ in H&N OARs^c^	18%
Single vs multi-field delivery per fraction	EQD2_mean_ in lung^d^	56%^e^
No vs daily adaptation	D_1cc_ in H&N OARs^f^	29%
	D_mean_ in H&N OARs^g^	15%
10 or 5 Gy vs 2 Gy/fraction for high α/β tumours	EQD2 in OAR region receiving prescribed dose^h^	56% or 28%^i^
Incomplete FLASH dose-rate coverage	Reduced FLASH sparing in lower dose-rate volumes^j^	0–60%

^a.^ Average of maximum dose increases in the oral cavity, mandible, spinal cord, brainstem, chiasm and left/right optic nerves, cochlea, parotids and lenses (20).

^b.^ Average of mean dose increases in the oesophagus, heart and spinal cord (21).

^c.^ Average of maximum dose increases in the oral cavity and left and right cochlea and parotids (20).

^d.^ Average mean EQD2 increase within the ipsilateral lung minus PTV (30).

^e.^ This mean EQD2 increase would be offset by a ~30% increase in isotoxic dose-level at UHDR, according to the LQ model (30).

^f.^ Average of increases in dose to the most highly irradiated 1 cc of cord and brainstem (22).

^g.^ Average of mean dose increases in the larynx, constrictor muscles, oral cavity and contralateral and ipsilateral parotids (22).

^h.^ Increase in normal tissue EQD2 when delivered using a hypofractionated schedule vs a 2 Gy schedule delivering the same tumour EQD2, calculated for normal tissue and tumour α/β values of 3 and 10 Gy.

^i.^ These EQD2 increases would be offset by 30% and 15% increases in isotoxic dose-levels at UHDR according to the LQ model (9).

^j.^ Roughly10% by volume of highly dosed regions of OARs receive< 40 Gy s^-1^, typically 20 Gy s^-1^ or less, potentially losing FLASH sparing (20, 25, 27).

Much of the gain from FLASH may be undone by using transmission rather than Bragg peak planning, or delivering one field per fraction, or using 5–10 Gy doses-per-fraction to treat tumours standardly given 2 Gy fractions, or not adapting delivery to reflect on-treatment images. For a series toxicity with incidence determined largely by the highest dose-level in an OAR rather than the volume irradiated, the FLASH gain might also be reduced or lost by failing to completely irradiate high-dose OAR regions at ≥40 Gy s^-1^. Thus, for proton FLASH to substantially improve outcomes, the challenges listed in the table need to be systematically overcome.

Individual losses in **Table 3** will be compounded if more than one compromise is made. However, compounding will be incomplete since different compromises affect different OARs. For example, transmission fields will primarily raise doses in OARs at some distance beyond the target. On the other hand, by using hypofractionated schedules for tumours standardly treated in 2 Gy fractions normal tissue EQD2s will be raised in all structures including those abutting the target.

### 3.2 Physics solutions

#### 3.2.1 Achieving FLASH dose-rates in Bragg peak treatments

Current Bragg peak FLASH research lies at the interface between plan optimization and ridge filter design. One optimization approach seeks to achieve range modulation using steps coarse enough to lie within the tolerance of 3D printers or milling machines used to build ridge filters, but fine enough to generate close-to-optimal dose-distributions (78).

Another approach aims to simultaneously achieve the best dose-distributions and most rapid FLASH irradiation of normal tissues (27). Serious complications such as perforation and stenosis of airways, blood vessels, oesophagus and intestine are considered series-like (79–82), and their incidence may be substantially increased if even small subvolumes of highly-dosed regions are irradiated at levels below the FLASH threshold. Thus, it should be a priority for plan optimization to achieve FLASH dose-rates comprehensively throughout high-dose regions of normal tissues in which series toxicities occur.

#### 3.2.2 Multi-field planning

**Figure 2:**
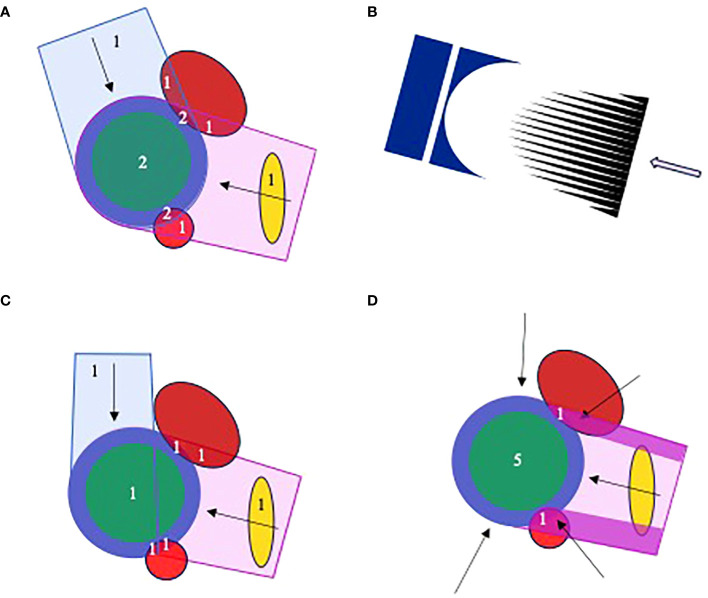
Proton planning for FLASH, illustrated for a schematic geometry with CTV (green), PTV (blue), two adjacent OARs (red) and a more distant OAR (gold). Numerals in the plots describe numbers of fields that irradiate different structures. **(A)** A 2-field plan in which the PTV and overlapping OAR are irradiated by both fields. **(B)** The cerise field in **(A)** is generated by scanning a pencil beam through a range modulator (black) and compensator (dark blue). **(C)** A 2-field plan patched distal-to-lateral edge, in which the adjacent OARs are irradiated by a single field. **(D)** A 5-field plan in which the adjacent OARs are irradiated by only the high fluence regions of one field, here the purple segments of the left lateral-posterior. The other fields and remainder of the first field are optimized to deliver a uniform dose throughout the PTV. The field irradiating the OAR-PTV overlap regions can be changed throughout treatment. The distant OAR will be irradiated by one or more fields, depending on field orientations.

The conflicting demands placed on the delivery of multi-field plans by FLASH and LQ considerations are illustrated in **Figure 2** for a schematic patient geometry that includes a CTV expanded to form a PTV, two adjacent OARs which overlap the PTV and a distant OAR.

**Figure 2A** shows a schematic two-field proton plan in which the PTV and overlapping subvolumes of adjacent OARs are irradiated by both fields, with the distant OAR irradiated by a single field. From the FLASH perspective, delivery of one field-per-fraction is best, allowing the OAR-PTV overlap regions to be irradiated within the 0.2–0.5 s limit thought necessary for FLASH sparing. According to the LQ model, though, this approach is less good than delivering both fields at each fraction, because it substantially varies the doses delivered to the distant OAR from one fraction to the next, increasing the EQD2.

These conflicting FLASH and LQ demands affect multi-field treatments irrespective of beam-type. However, the distal edge of proton beams suggests a particular solution. **Figure 2C** illustrates an initial patch planning approach (83–85). The figure shows a patch field with distant-to-lateral field edge matching. A ‘disjoint field’ technique based on lateral-to-lateral edge matching has previously been described (86). Both fields in the figure can be delivered at each fraction, with individual OAR-PTV overlap regions being irradiated by only one field. Thus, both LQ and FLASH considerations are addressed. However, each field in this plan delivers the whole prescribed dose only to a fraction of the PTV, whereas in **Figure 2A** each field delivers a fraction of the prescribed dose to the whole PTV. The physical dose delivered to the distant OAR will therefore be considerably higher for the plan in **Figure 2C** than in **Figure 2A**. And this counteracts the reduction in EQD2 relative to physical dose achieved by delivering both fields in **Figure 2C** at each treatment fraction.

An improved approach using segmented fields is shown schematically in **Figure 2D**. Here, the OAR-PTV overlap regions are irradiated entirely by segments of the left lateral-posterior field. Fluences delivered by other fields and the remainder of the first are optimized to uniformly irradiate the rest of the PTV. This allows all fields to be delivered at each fraction while irradiating OAR-PTV overlap regions through only one field and keeping doses to the distal OAR quite low. By varying the field used to irradiate overlapping OAR-PTV regions from fraction to fraction, doses along the high intensity segments can be diluted in the overall treatment. Effects of range uncertainties on matches of distal edges of other fields to lateral edges of the high intensity segments can similarly be diluted (84).

#### 3.2.3 Dynamic proton pull-back and range modulation

3D ridge filters are built as arrays of pins, each of which spreads out the Bragg peak to a width proportional to the range of lengths along the pin (**Table 4**). The ridge filters can be 3D-printed from plastics or milled from metals such as aluminium (18), but to make online adaptation feasible, dynamic proton range modulation devices are needed.

**Table 4:** Properties of pin ridge filters and some dynamic alternatives for creating proton range modulation.

Range modulator	Description	Advantages	Disadvantages	Diagram
Pin ridge filter	• Two-dimensional grid of pins of varying lengths • Spreads proton energies out to cover the target volume with dose • Used in conjunction with a range compensator	• Low manufacturing costs • Achieves FLASH dose-rates for Bragg peak deliveries • Suitable for weekly offline adaptation	• Slow manufacture – unsuitable for daily online adaptation • QA of every ridge filter may be required • Used together with a field-specific compensator • Increases neutron radiation compared to conventional proton beam scanning	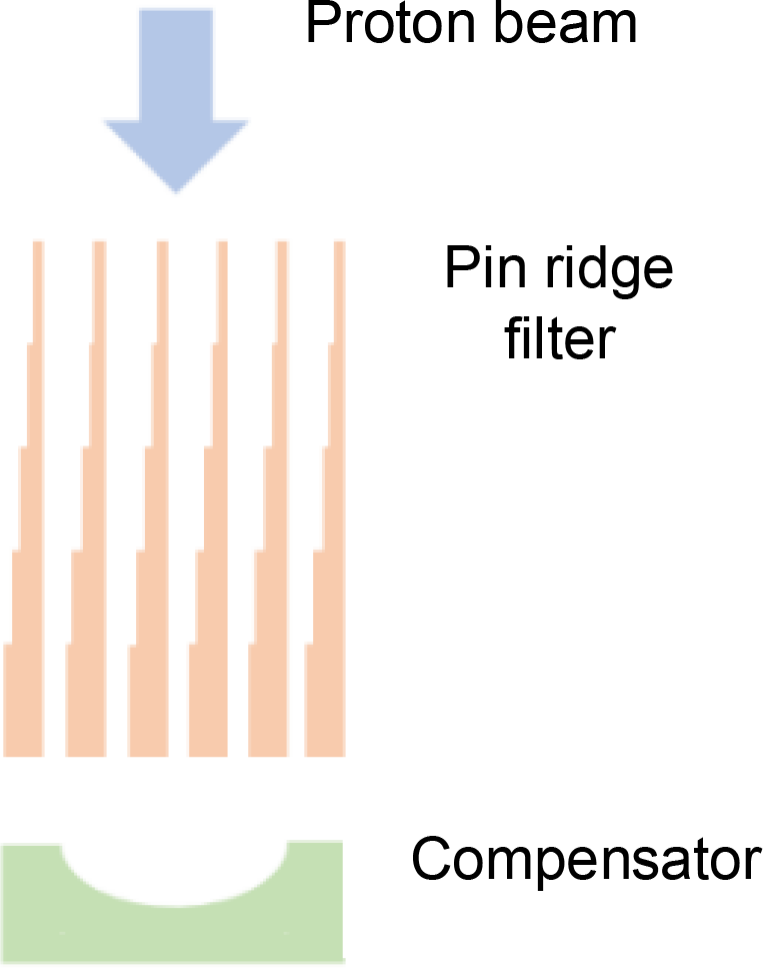
Universal ridge filter	• Superposition of two periodic layers with a variable offset • Triangular or more complex wave-trains • Trades off proton range modulation against pull-back	• Adjustable to account for anatomical variations throughout treatment • And for differences between patients • May not need to carry out QA for each filter setting	• Needs an adjustable compensator to offset changes in proton pull-back that accompany changes in range modulation • Increases neutron radiation	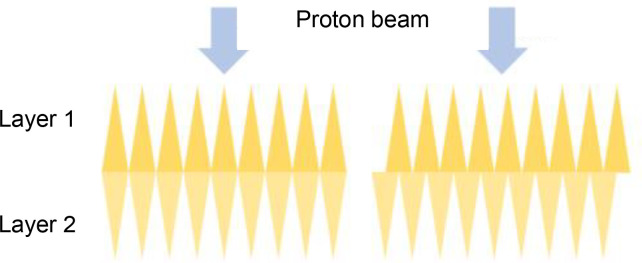
Rapidly switching energy degrader	• A stack of rapidly moving energy degraders could achieve range modulation at FLASH dose-rates • Would need to be located close to the patient to reduce beam losses • A dual wedge degrader might achieve similar results	• No need for a separate compensator • May not need to QA for each range modulation pattern	• Reproducible high-speed switching requires robust engineering • Likely needs regular system QA and maintenance * Increases neutron production	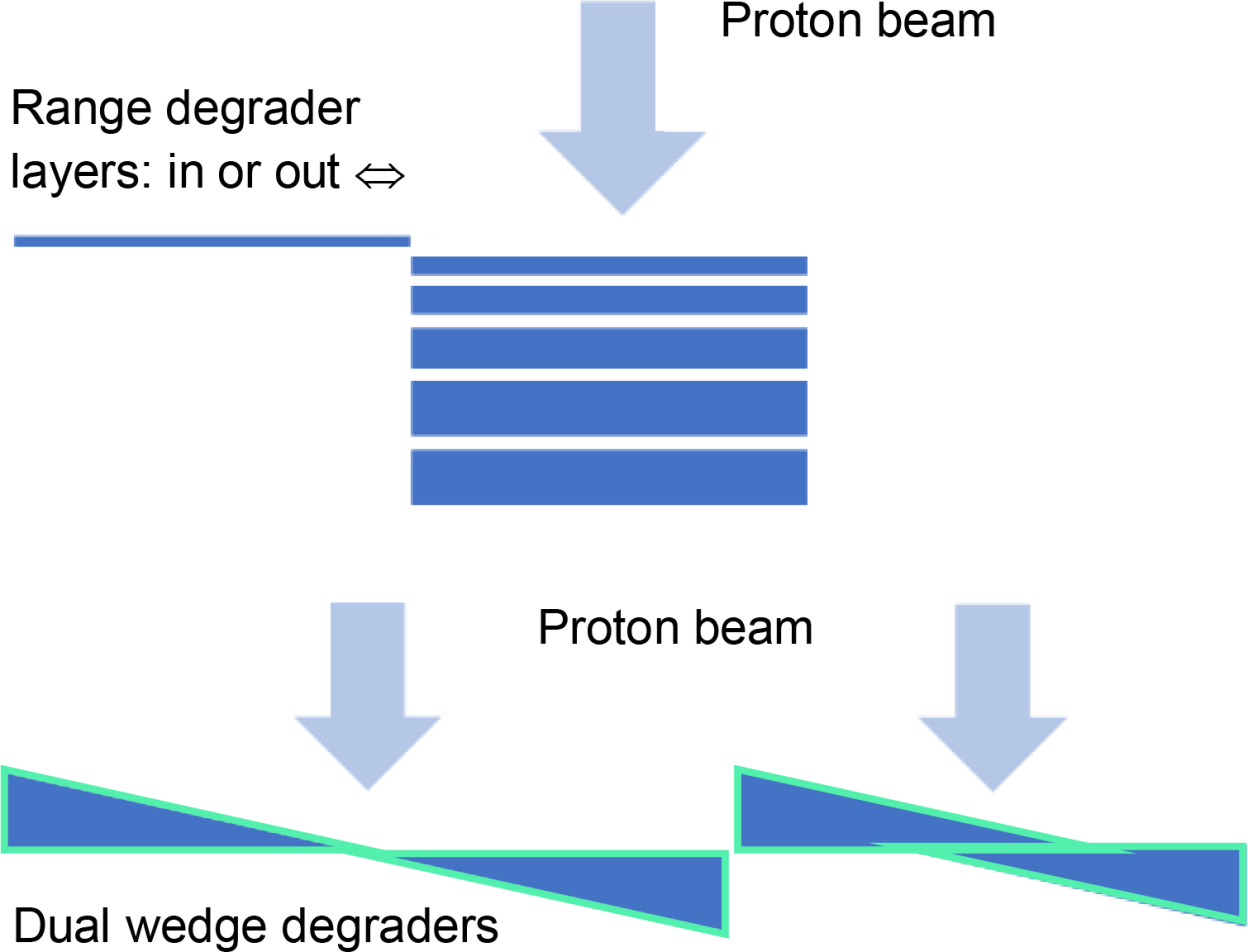

Some dynamic filter designs have already been proposed. Zhang et al. have described a system composed of a stack of multileaves (87). By varying the positions of leaf ends between layers of the stack, a range of summed leaf thicknesses is created, modulating the proton range. The modulation pattern can be adjusted by changing the leaf settings but is only defined in a limited region around the leaf ends. It is therefore intended that the leaves track the scanned proton pencil beam, which may require them travelling up to 10 ms^-1^ during FLASH treatments. The engineering challenges of achieving these speeds while guaranteeing accurate leaf positioning are considerable. For comparison, the maximum speeds of multileaves manufactured by Elekta (Crawley, UK) and Accuray (Madison, WI) are currently 0.08 and 2.5 ms^-1^.

Alternatively, Maradia et al. have proposed a universal dynamic ridge filter built by overlaying two identical, periodically repeating individual filter patterns (88). The concept was illustrated using two saw-tooth waveforms superimposed in-phase and anti-phase, achieving either twice the level of range-modulation generated by each layer alone with no pull-back, or no modulation but pull-back equal to the thickness of the saw-tooth pattern (**Table 4**). A more complex repeating shape which provided a flatter spread-out Bragg peak was used for the system’s practical realization.

The range modulation provided by this filter is spatially uniform but can in principle be varied as a pencil beam is scanned across it. The spatial period of the filter studied was 0.55 cm, allowing the range modulation to be changed from its minimum to maximum level by shifting one layer 0.275 cm. To achieve this in a 1 ms interval between delivery of two neighbouring pencil beams in a scanned FLASH treatment, the layer would need to move at 2.75 m s^-1^ or half that speed if both layers move.

Another solution would be a fast-switching range modulator with low beam losses. The *S250i* synchro cyclotron proton system (Mevion, Littleton, MA) has a range modulator located in its nozzle (89). It comprises 18 polycarbonate layers of varying thickness which can be combined to create 161 total thicknesses and associated proton energy reductions, as shown schematically in **Table 4**. The ~50 ms energy switching time might permit painting of a few energy layers within the 0.2–0.5 s duration limit for FLASH. The beam energy could alternatively be degraded by dual wedges with a variable offset. A dual wedge-based energy degrader in current use can change the energy from 230 to 70 MeV in<50 ms (90, 91). Conventional energy selection systems can also be made more efficient by using single wedges to reduce the momentum range of protons that have travelled through an energy degrader and dipole magnet, without incurring the beam losses that result from a momentum-selection slit (92).

A further option would be to construct ridge filter pins from individual pre-manufactured columns (78). Each filter might comprise several thousand columns, and so a rapid-assembly system would be required for online adaptation.

#### 3.2.4 Metrology solutions

Calorimetry is the most direct method for measuring absorbed dose, based on detecting temperature increases due to energy absorbed from ionizing radiation. Calorimeters have been used as primary dosimetry standards for many years, and since their sensitivities are independent of dose-rate, they are well-suited for UHDR beam dosimetry.

Due to their complex and bulky nature, calorimeters have traditionally been operated only at NMIs, with dosimetry in clinics being performed using cross-calibrated ionization chambers. However, the UK National Physical Laboratory (NPL) has now developed a portable primary-standard graphite calorimeter (PSPC) for direct use in clinical proton beams (93, 94).

For the *FAST-01* clinical trial, the UHDR proton beam was traceably calibrated using the PSPC. The overall calibration uncertainty was 0.9% (1σ), a level compatible with current clinical recommendations (95). Establishing the calibration within acceptable accuracy limits was a requirement for approval of this first in-human proton FLASH trial by the US Food & Drug Administration.

The UHDR proton beam employed in *FAST-01* was also measured using parallel plate ionization chambers calibrated at standard dose-rates, with recombination corrections determined using the two-voltage method. Readings from the Advanced Markus chamber (PTW, Freiburg, Germany) operated at 400 V with a 1.006 recombination correction factor agreed with PSCP dose measurements to within 0.2%. The PPC05 chamber (IBA, Louvain-la-Neuve, Belgium) over-read by 3% when operated at 400 V with 1.002 recombination correction (23).

Vented parallel plate ionization chambers with electrode separations of ~0.25 mm are promising detectors for real-time FLASH dosimetry. One design has collection efficiencies >99% at 250 V and doses-per-pulse up to 5.4 Gy, with negligible ion multiplication (96).

Developments in FLASH RT absolute dosimetry using calorimeters and ionization chambers have recently been reviewed by Subiel et al. (97). Dosimetry guidelines do not yet exist for FLASH in general (98), however the *FlashForward*™ Consortium is presently collating dose measurements made at FLASH-enabled proton therapy centres, with the aim of establishing guidelines for FLASH-PBT.

### 3.3 Clinical and pre-clinical solutions

#### 3.3.1 Tumour selection

The increased normal tissue EQD2s that result from giving FLASH schedules with doses-per-fraction ≥5–10 Gy to tumours best treated in 2 Gy fractions can be avoided by selecting tumours standardly treated using SABR/SRS or which have low α/β ratios. Tumours treated using SABR/SRS include early-stage lung cancer, pancreas cancer, renal cell carcinoma, liver malignancies, oligometastatic disease and several brain tumours. Low α/β tumours include prostate and breast cancers, rhabdomysarcoma, liposarcoma, melanoma, acoustic neuroma, meningioma and chordoma (99). Tumour selection for proton FLASH may be guided by policies for standard PBT (100). However, FLASH may offer notable improvements over standard RT, and deep-seated tumours can currently only be treated at UHDR using proton beams. Therefore, the range of indications may be wider for proton FLASH than for standard proton treatments.

Recently, substantial FLASH sparing was reported for ten daily fractions of 3 Gy given at ultra-high and standard dose-rates, assessed using an electro-physiological measure of synaptic plasticity in mice (71). This raises the prospect that FLASH sparing might be achieved in the clinic using moderately hypo-fractionated schedules and perhaps even 2 Gy fractions, extending the range of tumour sites suitable for FLASH.

#### 3.3.2 Pre-clinical FLASH characterization

Translation of findings from pre-clinical to clinical RT has often been frustrated by the limited clinical relevance of pre-clinical cancer models, treatment schedules and endpoints studied (101). To avoid repeating this experience for proton FLASH, pre-clinical experiments carried out ahead of clinical studies should be closely related to proposed treatments, as summarized in the first two columns of **Table 5**.

**Table 5:** Further pre-clinical characterization to inform proton FLASH treatment design.

Test clinically related endpoints	Test temporal profiles of proton treatments	Refine irradiation thresholds for FLASH sparing
• Measure changes in normal tissue structure and function reflecting toxicity profiles of proton RT • Use longer-term measures of tumour control • Study low and high α/β tumours	• Use pulsed irradiation time-structures similar to scanned proton fields • Introduce gaps between irradiation segments to reflect ~1 minute intervals between field deliveries	• How low can the dose-per-fraction be? • How low can the dose-rate be? • How long can irradiation take?

Endpoints should be chosen to represent a range of relevant treatment outcomes. Taking lung RT as an example, it would be informative to pre-clinically test FLASH sparing of lung, oesophageal and cardiac damage. Tumour response studies should include long-term measures of control and assess whether FLASH sparing is absent in both low and high α/β tumours. Effects of temporal features imposed by FLASH proton delivery systems should also be tested, including pulsed irradiation during delivery of individual fields and time intervals between fields.

Studies should be carried out to improve the precision with which FLASH sparing thresholds are known, investigating the minimum dose-per-fraction and dose-rate and maximum irradiation duration needed, as summarized in the final column of **Table 5**. For example, if FLASH sparing is achievable using 2 Gy fractionation, high α/β tumours could be treated without the normal tissue EQD2 increases that result from hypofractionation.

These points are starting to be addressed. Pre-clinical studies have assessed FLASH sparing for pneumonitis and lung fibrosis (60–62); used a scanned proton beam with a time-structure similar to clinical FLASH proton fields (53); and measured the FLASH effect for fractions as small as 3 Gy (71). Such experiments allow key features of treatments to be pre-clinically tested ahead of clinical studies, to check treatment design and trial powering.

## 4 Discussion

FLASH RT may transformatively widen the therapeutic window between tumour killing and damage to neighbouring normal tissues. However, for FLASH sparing to be achieved it is currently thought that the dose-rate should be ≥40 Gy s^-1^, the irradiation duration ≤0.2–0.5 s and dose-per-fraction ≥5–10 Gy (7, 9, 10).

Proton accelerators are presently the only clinical RT systems capable of UHDR irradiation of deep-seated tumours. Even using cyclotron-based systems, though, it is challenging to meet the FLASH conditions, and this potentially leads to several treatment compromises. These include using transmission beams rather than Bragg peak fields to create plans (16); giving single fields at each fraction (30); not adapting Bragg peak plans online because ridge filters have lengthy manufacturing times (17–19); prescribing doses-per-fraction ≥5–10 Gy to tumours that are better treated using standard 2 Gy fractionation (9); and accepting dose-rates below the FLASH threshold in small subvolumes of highly-dosed normal tissues (20, 25, 27).

While some pre-clinical experiments have reported large radioprotective effects for ultra-high dose-rates, many have reported more moderate effects with FLASH dose gains typically being 5–20% (11). Results from planning studies suggest these moderate gains would be substantially reduced or nullified by some of the compromises listed in **Table 5** if made individually, and might be reversed if multiple compromises were made.

Alongside the challenges for delivering proton FLASH, we have described possible solutions. These include using 3D ridge filters for Bragg peak delivery (17, 18, 20, 27); creating multi-field plans in which critical normal tissues are irradiated by only a single field; building dynamic systems for proton range modulation and pull-back that allow online plan adaptation (87–89); selecting tumours that are standardly treated using SABR/SRS or have low α/β ratios; and developing planning algorithms that simultaneously optimize dose-rates and doses, aiming to achieve comprehensive UHDR coverage of highly-dosed structures in which series toxicities arise (27).

Prospects of success for proton FLASH will be improved by carrying out further pre-clinical characterization for endpoints closely related to toxicity and survival in clinical treatments. Doses-per-fraction being considered clinically should be tested, as should the irradiation time-structures of proton FLASH treatments. Data are also needed to define more precisely how low the dose-rate and dose-per-fraction can be while achieving FLASH sparing, and how long irradiation duration can be.

In conclusion, proton beam FLASH may profoundly improve RT outcomes by protecting normal tissues neighbouring tumours from the effects of high-dose irradiation, a step not achieved by successive developments in RT technology. However, the stringent conditions thought necessary to achieve FLASH dose sparing make new and disruptive demands on treatment delivery. A series of technological and radiobiological advances are therefore needed to realize the potential of FLASH-PBT.

## Author contributions

JF: Conceptualization, Funding acquisition, Investigation, Methodology, Supervision, Visualization, Writing – original draft, Writing – review & editing. CM: Investigation, Visualization, Writing – original draft, Writing – review & editing. SJ: Investigation, Writing – original draft, Writing – review & editing. RA: Conceptualization, Investigation, Writing – original draft, Writing – review & editing. MH: Conceptualization, Funding acquisition, Investigation, Supervision, Writing – original draft, Writing – review & editing.
